# Bacteroides, Metabolites, and Lymphocytes: A Triad of Biomarkers in Small Intestinal Bacterial Overgrowth

**DOI:** 10.1002/fsn3.70367

**Published:** 2025-06-20

**Authors:** Ziteng Wang, Wentao Tan, Lan Wu, Pengfei Zhang, Huanhuan Xiong, Licun Zhu, Jiali Huang, Jianfang Cui, Li Li, Chunmei Guo, Lingling He, Hongshan Wei, Hong Liu

**Affiliations:** ^1^ Gastroenterology Department, Beijing Shijitan Hospital Capital Medical University Beijing China; ^2^ Gastroenterology Department, Beijing Ditan Hospital Capital Medical University Beijing China; ^3^ Design Institute Chinergy Limited Company Beijing China; ^4^ Emergency Department, Beijing Tiantan Hospital Capital Medical University Beijing China; ^5^ Rheumatology and Immunology Department Zhumadian Central Hospital Henan China; ^6^ Gastroenterology Department, Shougang Hospital Peking University Beijing China

**Keywords:** clinical symptoms, hydrogen methane breath test, intestinal flora, lymphocyte count, small intestinal bacterial overgrowth

## Abstract

The purpose of this study was to investigate the clinical characteristics of small intestinal bacterial overgrowth (SIBO) and analyze its relationship with changes in intestinal microbiota and metabolites. The study included patients from Beijing Shijitan Hospital (Feb 2021–Nov 2023) who underwent hydrogen‐methane breath tests, hematological tests, and completed questionnaires. Baseline characteristics of SIBO patients were analyzed using descriptive statistics and independent t‐tests. Logistic regression and Receiver Operating Characteristic (ROC) curve analysis identified potential SIBO predictors. Chi‐square tests assessed the association between SIBO and symptoms. Stool samples from 29 patients underwent 16S rRNA sequencing to analyze gut microbiota and metabolites. Then we explored links between microbiota, metabolites, symptoms, and serum markers. 405 persons in all completed the hydrogen methane breath test, of whom 109 (26.91%) were in the SIBO− and 296 (73.09%) were in the SIBO+. No significant differences were observed between the SIBO+ and SIBO− groups in terms of gender, age, or BMI. However, lymphocyte counts were significantly different between the groups, with lymphocyte count proving to be a better predictor of SIBO in middle‐aged and elderly non‐obese men. SIBO was significantly associated with diarrhea and acid reflux symptoms. 16S rRNA sequencing of stool samples from 29 patients revealed that *Bacteroides* was the predominant intestinal microbiota in SIBO patients. Metabolic pathways involving various metabolites were identified as significantly altered in these patients. Additionally, there were significant correlations between various intestinal flora and metabolites with serum markers.

## Graphical abstract

We identified lymphocyte count as a potential predictor of SIBO in middle‐aged and elderly non‐obese men. Additionally, our findings revealed significant associations between SIBO and symptoms such as diarrhea and acid reflux, as well as alterations in gut microbiota—specifically the predominance of Bacteroides—and metabolic pathways.

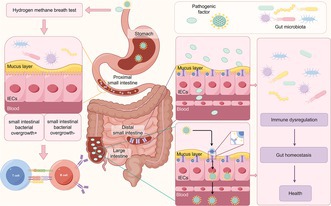

## 1 Introduction

Small Intestinal Bacterial Overgrowth (SIBO) is a pathological condition defined by an excessive proliferation of bacteria in the small intestine, accompanied by an alteration in the normal composition and diversity of the microbial community (Banaszak et al. 2023). Common etiological factors of SIBO include structural abnormalities of the intestine, intestinal dysmotility, deficiencies in antimicrobial defense mechanisms, and dyscoordination between the small and large intestines. These factors can interfere with the normal processes of digestion and nutrient absorption, leading to clinical symptoms such as diarrhea, steatorrhea, abdominal pain, bloating, flatulence, and malnutrition (Banaszak et al. 2023). Beyond exacerbating symptoms of dyspepsia, SIBO can also contribute to metabolic disturbances and immune dysfunction. The clinical diagnosis of SIBO can be challenging due to its non‐specific symptoms. The gold standard for SIBO is the collection and bacterial culture of a small intestine fluid sample, which allows for the identification and quantification of bacterial species present. However, this invasive approach limits its practical application in clinical settings. Alternatively, the hydrogen‐methane breath test has become widely recommended by the American College of Gastroenterology as a non‐invasive diagnostic tool (Quigley et al. 2020). The test measures the concentrations of hydrogen (H_2_) and methane (CH_4_) in their exhaled breath over time. Elevated concentrations of either H_2_ or CH_4_ suggest the potential presence of SIBO (Maeda and Murakami 2023).

SIBO significantly impacts gut health through several interrelated mechanisms. First, SIBO can disrupt intestinal motility, resulting in either reduced or disordered motility. This dysfunction causes food and bacteria to linger in the small intestine for prolonged periods, promoting bacterial accumulation and overgrowth, which in turn exacerbates the imbalance in the microbiota (Van Citters and Lin 2005). Second, SIBO can also activate the immune system, triggering both local and systemic inflammatory responses. The overgrown bacteria and their metabolites—such as endotoxins and short‐chain fatty acids (SCFAs)—stimulate the intestinal immune system, leading to the release of pro‐inflammatory cytokines. This chronic, low‐grade inflammation not only compromises intestinal barrier function but also has the potential to affect immune and neurological functions through the gut‐brain axis (Riordan et al. 1998). Additionally, SIBO interferes with the digestion and absorption of carbohydrates in the small intestine. As bacteria proliferate, they consume undigested food, particularly carbohydrates, and produce gases such as H_2_ and CH_4_. Then, the gases contribute to symptoms like bloating and abdominal pain while also impairing the absorption of essential nutrients. Prolonged malabsorption can result in nutrient deficiencies, further exacerbating gastrointestinal and systemic health issues (Posserud et al. 2007). Moreover, SIBO has a notable impact on bile acid metabolism. The overgrowth of intestinal bacteria alters the transformation processes of bile acids, which may lead to abnormal bile acid concentrations. This disruption can impair the digestion and absorption of fats, potentially causing symptoms like steatosis (Song et al. 2020). Abnormal bile acids can also negatively affect the diversity and stability of the gut microbiota, further exacerbating intestinal dysbiosis (Losurdo et al. 2020). Perhaps most importantly, SIBO can lead to a condition known as “leaky gut” by compromising the permeability of the intestinal epithelial barrier. Bacteria and their metabolites breach the intestinal wall, impairing barrier function and allowing harmful substances and pathogens to enter the bloodstream. This results in a systemic inflammatory response. The destruction of the intestinal barrier not only increases the risk of food allergies but may also lead to immune system dysregulation, ultimately impacting overall health (Gudan et al. 2023). In summary, SIBO contributes to intestinal dysbiosis through a combination of mechanisms, including disruption of intestinal motility, immune responses, digestion and absorption processes, and bile acid metabolism. The interaction of these factors exacerbates intestinal dysfunction and may lead to more complex health issues affecting multiple systems in the body, including metabolic, immune, and neurological functions. Therefore, understanding the mechanisms underlying SIBO and its impact on gut health is crucial for early diagnosis, intervention, and treatment.

Currently, research on SIBO has primarily focused on its associations with various diseases, particularly metabolic, gastrointestinal, and neuropsychiatric disorders (Sroka et al. 2022). However, while these studies have significantly advanced our clinical understanding of SIBO, there remains a gap in fully exploring how SIBO affects the structure and composition of the small intestinal bacterial community. Most existing research has centered on the clinical presentation of SIBO and the development of diagnostic tools, without adequately addressing the specific characteristics of the intestinal microbiota imbalance following SIBO infection, particularly in terms of bacterial species, abundance, and how microbiota interactions influence host health. Although a strong association between SIBO and gut microbiota disorders has been established, systematic analyses of how specific microbiota, especially those in the small intestine, interact with host immune, metabolic, and digestive functions are still lacking. The role of small intestinal flora in SIBO may further influence overall host health by regulating critical physiological processes such as immune responses, carbohydrate metabolism, and bile acid metabolism. However, the underlying microscopic mechanisms of these changes remain poorly understood, and further experimental data and detailed studies are necessary to bridge this knowledge gap. To address this, our research aims to fill this gap by thoroughly describing the clinical characteristics of SIBO and investigating the specific gut microecological mechanisms involved. We seek to identify the key microbial communities that play an important role in the onset, progression, and disease associations of SIBO. Using advanced technologies such as high‐throughput 16S rRNA gene sequencing, we will conduct a comprehensive and systematic analysis of the gut microbiota in SIBO patients to uncover the characteristics of microbiota changes. Furthermore, these microecological investigations will not only deepen our understanding of the basic pathological mechanisms of SIBO but also provide new biomarkers and potential therapeutic targets for clinical practice. This research will ultimately offer a more solid scientific foundation for the accurate diagnostic methods and personalized treatment strategies for SIBO.

## 2 Method

### 2.1 Study Population

Between February 2021 and November 2023, we selected individuals who had undergone lactulose hydrogen‐methane breath tests. A total of 405 patients completed these tests. The patients were categorized into the SIBO group and the non‐SIBO group. Then, stool samples from patients who completed these tests between July 2023 and November 2023 were collected for gut microbiota sequencing analyses.

#### 2.1.1 Inclusion Criteria

- Subjects older than 18 years.
- Were subjected to a methane hydrogen breath test.
- Completed a questionnaire and provided comprehensive medical records.

#### 2.1.2 Exclusion Criteria

Patients were excluded from the study based on the following criteria:

- Gastrointestinal or Abdominal Surgery: A history of gastrointestinal surgery, abdominal surgery, or any surgery affecting the gastrointestinal system (e.g., gastric bypass, colectomy).
- Renal or Infectious Diseases: A history of renal insufficiency or other infectious diseases that could impact gastrointestinal function.
- Comorbidity: including but not limited to diseases that may affect gastrointestinal function, such as Crohn's disease, ulcerative colitis, cirrhosis of the liver, hypothyroidism, intestinal obstruction, etc.
- Recent medication use: Use of antibiotics (e.g., amoxicillin, clarithromycin) or drugs that may affect gastrointestinal function (e.g., domperidone) within the past month.
- Recent Gastrointestinal Issues: A history of enema treatment or acute enteritis in the past week, or malabsorption, or other gastrointestinal diseases.
- Pregnancy and Lactation: lactating women or Pregnant.
- Intolerance: A known history of lactose intolerance.
- Study Protocol Non‐compliance: Persons who do not conform to the study protocol, fail to provide informed consent, have not completed personal information that may impact the results, or are unable to fully cooperate with the study procedures.

## 3 Data Acquisition and Research Methods

### 3.1 Collection of Basic Data

531 persons in all underwent the hydrogen‐methane breath test, and after applying inclusion and exclusion criteria, 405 participants were included in this retrospective study. Lifestyle factors, such as smoking and drinking habits, as well as medical histories, including chronic conditions like hypertension, diabetes, metabolic syndrome, and cardiovascular disease, were documented. Demographic data, including age, sex, height, weight, and body mass index (BMI), were recorded, with BMI = weight (kg)/height squared (m^2^). Additionally, histories of abdominal surgery and digestive disorders (e.g., gastritis, ulcers) were noted. Participants responded to a comprehensive questionnaire, which included clinical symptoms such as gastroesophageal reflux, diarrhea, abdominal pain, constipation, and flatulence.

Following a 6‐ to 12‐h fast, 1–2 mL of venous blood was drawn in the morning following a 6‐ to 12‐h fast or at rest, and the samples were subsequently stored in anticoagulant tubes for analysis. Blood tests included hematological indicators, liver function markers, metabolic markers, and electrolytes. All tests were performed using the XS‐1000i system (Roche Diagnostics, Shenzhen, China) to ensure precision and reliability. This comprehensive data collection protocol enabled the detailed analysis of clinical characteristics and laboratory markers within the study cohort.

### 3.2 Examination Methods and Diagnostic Criteria

#### 3.2.1 Lactulose H_2_ ‐CH_4_ Breath Test

Participants were required to follow a standardized preparation protocol before undergoing the lactulose hydrogen‐methane breath test (Quintron Corporation, United States) to ensure accurate and reliable results. The protocol consisted of the following steps:

- Fasting Requirement: Participants were instructed to fast for a period of 6 to 12 h before the test to eliminate any potential interference with the results.
- Dietary Restrictions: Person was advised to avoid foods that are slow to digest, high in fiber, or known to produce excess H_2_, for example, pasta, beans, and cereals. Additionally, overeating was discouraged to ensure that digestive processes would not affect the outcomes.
- Medication Restrictions: Person was instructed to refrain from using sedatives or sleep aids on the day preceding the test to prevent any interference with the testing process.
- Smoking Prohibition: Refrain from smoking for at least 1 h prior to the test to prevent any potential impact on the results.
- Oral Hygiene: Prior to the test, persons were required to thoroughly brush their teeth, rinse their mouths, and maintain proper oral hygiene to avoid any contaminants that could influence the test outcome.

##### 3.2.1.1 Test Procedure

Participants began the breath test procedure by taking several deep, steady breaths to ensure a relaxed state. Then let them take a deep breath and slowly exhale into the air bag. The first breath air served as the baseline measurement. Following the baseline measurement, participants were orally administered 10 mL of lactulose solution (Abbott, Netherlands), and the timing for the test commenced immediately. To ensure accurate results, participants provided subsequent breath samples at 30‐min intervals, resulting in a total of six samples. Each exhalation was required to completely fill the designated collection bag to ensure adequate sample volume. To minimize bacterial contamination and interference with test results, participants were instructed to rinse their mouths thoroughly after consuming the lactulose solution.

##### 3.2.1.2 SIBO Positive Diagnostic Criteria

Based on the 2020 American College of Gastroenterology consensus, the diagnostic criteria for SIBO positivity are as follows (Quigley et al. 2020):

- H_2_ levels reached ≥20 ppm from baseline within 90 min after oral lactulose administration.
- CH_4_ levels were ≥ 10 ppm at any time point.

#### 3.2.2 Methods for Fecal Sample Collection and Analysis

Propensity score matching was employed to select a cohort of 30 hospitalized patients, maintaining a 1:2 ratio of SIBO− to SIBO+ individuals. However, due to the presence of additional complications in one case, this patient was excluded from the final analysis. As a result, the final cohort consisted of 29 patients, with 10 in the SIBO− group and 19 in the SIBO+ group. Fecal samples were collected from all participants using standardized fecal collection kits (fecal tube 76 × 20mm; Sarstedt Ag & Co. Kg, Germany). The samples were stored at −20°C within 2 h of collection and subsequently transferred to a −80°C freezer within 24 h to ensure optimal preservation. Ethical approval for the study was granted by the Ethics Committee of Beijing Shijitan Hospital, affiliated with Capital Medical University (Approval number: sjtky11‐1x‐2022 (063)).

##### 3.2.2.1 DNA Extraction and 16S rRNA Gene Analysis

Total fecal samples were extracted from fecal specimens using the TIANamp Fecal DNA Kit (TIANGEN, China). The integrity of DNA extracts was verified through 1% agarose gel electrophoresis, while DNA concentration and purity (A260/A280 ratio) were determined using a Nanodrop2000 ultramicrospectrophotometer (Thermo Scientific, USA).

The hypervariable V3‐V4 region of bacterial 16S rRNA genes was amplified in triplicate reactions using the ABI GeneAMP 9700 PCR system with universal primers 341F (5′‐CCTACGGGNGGCWGCAG‐3′) and 806R (5’‐GGACTACHVGGGTWTCTAAT‐3′). The thermal cycling conditions included an initial denaturation at 95°C for 3 min, followed by 30 cycles of denaturation at 95°C for 30 s, annealing at 55°C for 30 s, and extension at 72°C for 45 s. A final elongation step was carried out at 72°C for 8 min.

Sequencing libraries were prepared using the TIANSeq Rapid DNA Library Preparation Kit (Tiangen Biotechnology) following Illumina platform specifications. Library quality control included fluorometric quantification with a Qubit2.0 system (Thermo Scientific) and fragment size distribution analysis using an Agilent Bioanalyzer 2100. Paired‐end sequencing (2 × 250 bp) was subsequently performed on an Illumina platform.

Bioinformatic processing was implemented through the QIIME 2 pipeline (v2020.6). The raw sequencing data were first demultiplexed using the demux plugin, followed by primer removal using cutadapt. Sequence quality filtering, error correction using DADA2's denoising algorithm, read pair merging, and chimera elimination were systematically performed. Taxonomic classification was achieved through phylogenetic alignment against the Greengenes_13_8 reference database (99% OTU clustered version) within the QIIME 2 framework.

##### 3.2.2.2 Data Analysis and Reconstruction of Microbial Communities

The Short Multiple Regions Framework (SMURF) method was applied to reconstruct microbial communities using 16S rRNA gene sequencing data. This approach combines sequencing information from multiple amplified regions, which improves the precision of species identification and community analysis. SMURF begins with the preprocessing of high‐throughput sequencing data, which includes filtering out low‐quality sequences to maintain data integrity. Following this, k‐mers—short nucleotide fragments that overlap between the amplified regions and reflect the microbial community—are extracted. The Expectation–Maximization (EM) algorithm is subsequently applied to analyze the k‐mers and predict the most probable species composition within the microbial community. Species classification for each sequence is determined using a majority voting approach. The final results of the SMURF analysis provide both species identification and their relative abundance within each group. These outputs form the basis for further exploration of the microbial community's structure and diversity, offering robust and accurate insights into the microbial ecosystem (Nejman et al. 2020; Mishra et al. 2021; Parhi et al. 2020).

##### 3.2.2.3 Analysis of Metabolomic Profiles

Human fecal samples (100.0 mg ± 1.0 mg) were accurately weighed into pre‐cooled centrifuge tubes. To these, zirconium beads (3 mm in diameter) and 500 μL of pre‐cooled extraction solvent (methanol: acetonitrile: water = 2:2:1, v/v) containing 1 μg/mL of deuterosuccinic acid‐D4 internal standard were added. The cells were lysed using a three‐step method: Vortex oscillation for 30 s at 2500 rpm; Homogenization using a liquid nitrogen‐precooled tissue grinder for 4 min at 35 Hz; Ultrasonic crushing in an ice‐water bath for 5 min (40 kHz, 500 W). This process was repeated three times to ensure complete metabolite extraction. Following this, the protein precipitate was stored at −40°C for 1 h. The sample was then centrifuged at 13,800 × g at 4°C for 15 min (rotor radius 8.6 cm). The supernatant was filtered through a 0.22 μm nylon filter membrane and transferred to a specialized LC–MS sample vial.

Chromatographic separation was performed using a Thermo Vanquish UHPLC system, coupled with a Waters ACQUITY UPLC BEH Amide column (2.1 × 50 mm, 1.7 μm) maintained at 40°C. The mobile phase consisted of: (A) 25 mmol/L ammonium acetate (pH 9.75, adjusted with ammonia); (B) Acetonitrile. The gradient elution protocol was as follows: 0–1 min: 95% B; 1–8 min: 95% → 60% B; 8–9 min: 60% → 40% B; 9–10 min: 40% B; 10–10.1 min: 40% → 95% B; 95% B maintained until 12 min. The flow rate was 0.3 mL/min, with an injection volume of 2 μL. The temperature of the automatic injector was set to 4°C. Mass spectrometry was performed on a Thermo Orbitrap Exploris 120 high‐resolution mass spectrometer equipped with a HESI ion source. The ionization parameters for positive and negative ion switching mode were as follows: Sheath gas flow: 50 Arb; Auxiliary gas flow: 15 Arb; Ion transfer tube temperature: 320°C; Spray voltage: ±3.8 kV (positive), −3.4 kV (negative). The full scan range was m/z 70–1050, with a resolution of 60,000. Data‐dependent acquisition (DDA) mode was used to trigger second‐order fragmentation (HCD collision energy of 20/30/40 eV) at an MS/MS resolution of 15,000. The mass accuracy was ensured to be < 2 ppm through real‐time calibration.

The raw data were transformed into mzXML format using ProteoWizard, after which peak extraction, alignment, and integration were conducted with XCMS (v3.16.1), with signal‐to‐noise ratio (SNR) set at 3, alignment bandwidth (bw) at 5, and minimum fraction (minfrac) set at 0.5. Metabolite identification was performed based on the BiotreeDB database (v3.0), with metabolite annotations requiring matching of the exact mass (with mass deviation < 5 ppm), retention time, and secondary mass spectrum fragments (similarity > 80%). The data were preprocessed using PQN normalization and UV scaling.

### 3.3 Bioinformatics and Statistical Analysis

#### 3.3.1 Basic Information Statistical Analysis

Statistical analyses were conducted using SPSS software (version 23.0, IBM Corp., Armonk, NY, USA). The normality of the data distribution was evaluated using the Kolmogorov‐Smirnov (K‐S) test. Continuous variables are expressed as mean ± standard deviation (SD), while categorical variables are presented as percentages. Independent *t*‐tests were used to compare continuous variables between groups, and Fisher's exact test was applied for categorical variables. The prevalence of digestive symptoms was compared between SIBO‐negative and SIBO‐positive groups using the Chi‐square test. To examine the association between variables and SIBO status, multivariable‐adjusted odds ratios (OR) with 95% confidence intervals (CI) were computed through logistic regression, while multiple linear regression was utilized for continuous outcomes. Receiver operating characteristic (ROC) curves were constructed to assess the discriminatory performance of the model. The area under the curve (AUC) and its corresponding 95% confidence interval were calculated using the DeLong method. The optimal cutoff value was determined by maximizing Youden’s index. A *p*‐value of less than 0.05 (two‐tailed) was considered statistically significant for all analyses.

#### 3.3.2 Microbiota Analysis

The analysis was conducted based on 16S rRNA gene sequencing data. Operational Taxonomic Unit (OTU) clustering (97% similarity threshold) and species annotation were performed using the QIIME2 pipeline (v2020.6) with the Silva database (v138) for taxonomic assignment. Diversity analysis was carried out using R software (v3.6.2), following these steps: (i) Alpha diversity was evaluated using the following indices: Observed species, Chao1 index (species richness), Shannon index (species diversity), and Simpson index (species dominance). (ii) Beta diversity was assessed based on the Bray‐Curtis distance matrix. Principal coordinate analysis (PCoA), principal component analysis (PCA), and orthogonal partial least squares discriminant analysis (OPLS‐DA) were performed to visualize group differences. Permutational Multivariate Analysis of Variance (PERMANOVA) was used to assess the significance of differences between the three groups. (iii) Linear discriminant analysis effect size (LEfSe) was applied to identify differential taxa between groups. The Linear Discriminant Analysis (LDA) score threshold was set at 4.0, and significant taxa were identified using the Kruskal‐Wallis rank‐sum test. Effect sizes were also calculated for these differences. The analysis was performed on the Lianchuan Biotechnology Cloud Platform (Hangzhou Lianchuan Biotechnology Co. Ltd., ICP No. 150250535‐4).

For metabolite analysis, PCA, PCoA, and OPLS‐DA were conducted using the SIMCA‐P (v14.1) software. The criteria for identifying differential metabolites included: (i) a variable importance in projection value greater than 1.0; (ii) a Student's *t*‐test *p*‐value less than 0.05; and (iii) a fold change of at least 2.0. Metabolite annotation was performed using the Kyoto Encyclopedia of Genes and Genomes (KEGG) (Release 101.0), Human Metapolome Database (HMDB) (v5.0), and Lipid Maps databases. Enrichment analysis of metabolic pathways was conducted using the hypergeometric test, with a significance threshold of *p* < 0.05. To evaluate the correlations between bacteria and metabolites, Spearman's rank correlation coefficient was used. Data visualization was performed via the Metware Cloud platform (https://cloud.metware.cn/), generating volcano plots and integrated heatmaps (Lyu et al. 2023; Allaire et al. 2018).

## 4 Result

### 4.1 Retrospective Study Results

#### 4.1.1 Basic Clinical Demographic Characteristics of SIBO

A total of 405 patients completed the breath test, and the study compared basic information and clinical data between the SIBO− and SIBO+ groups (Table 1). The average age of the SIBO− group was 53.84 ± 14.05 years, while the SIBO+ group had an average age of 51.86 ± 15.86 years, with a *p*‐value of 0.227, indicating no statistically significant difference. Gender distribution showed 12.84% females and 14.07% males in the SIBO− group and 36.79% females and 36.30% males in the SIBO+ group, with a *p*‐value of 0.640, indicating no significant gender difference. The BMI of the two groups was 24.18 ± 5.40 kg/m^2^ in the SIBO− group and 24.52 ± 5.93 kg/m^2^ in the SIBO+ group, with a *p*‐value of 0.092, suggesting no significant difference. Smoking and drinking habits, as well as the incidence of diabetes and hypertension, were not significantly different between the two groups.

**TABLE 1:** Baseline clinical characteristics.

	Total	SIBO−	SIBO+	*p*
Age (years)	53.311 ± 14.567	53.844 ± 14.052	51.861 ± 15.863	0.227
Gender, *n* (%)	Female	52 (12.84%)	149 (36.79%)	0.640
	Male	57 (14.07%)	147 (36.30%)	
BMI (kg/m2)	24.269 ± 5.532	24.181 ± 5.396	24.520 ± 5.930	0.092
WBC (×10^9^ /L)	6.014 ± 1.862	6.106 ± 2.000	5.774 ± 1.424	0.184
Lymphocyte (×10^9^/L)	1.901 ± 0.596	1.939 ± 0.587	1.799 ± 0.612	**0.035**
Neutrophil (×10^9^/L)	3.545 ± 1.581	3.548 ± 1.554	3.555 ± 1.659	0.969
Hemoglobin (g/dL)	141.120 ± 15.676	138.172 ± 19.586	148.858 ± 12.492	0.359
Platelet (×10^9^/L)	225.310 ± 59.374	225.900 ± 60.260	223.770 ± 57.226	0.740
Monocyte (×10^9^/L)	0.361 ± 0.272	0.370 ± 0.308	0.338 ± 0.133	0.279
ALT (U/L)	22.182 ± 14.312	21.946 ± 13.682	22.798 ± 15.890	0.589
AST (U/L)	20.547 ± 9.187	20.330 ± 8.704	21.124 ± 10.380	0.434
GGT (U/L)	28.758 ± 27.349	29.171 ± 27.403	27.649 ± 27.298	0.617
Albumin (g/dL)	42.065 ± 4.456	42.163 ± 4.653	41.805 ± 3.896	0.468
UA (umol/L)	337.540 ± 90.605	334.741 ± 89.543	345.064 ± 93.401	0.310
Creatinine (umol/L)	68.190 ± 15.277	67.216 ± 14.311	70.843 ± 17.437	**0.035**
TC (mmol/L)	6.382 ± 2.417	7.037 ± 2.701	4.570 ± 1.044	0.392
TG (mmol/L)	1.460 ± 0.812	1.476 ± 0.809	1.416 ± 0.821	0.552
Glu (mmol/L)	5.691 ± 1.936	5.770 ± 2.135	5.479 ± 1.238	0.222
HDL‐C (mmol/L)	1.173 ± 0.311	1.176 ± 0.323	1.165 ± 0.276	0.786
LDL‐C (mmol/L)	3.003 ± 0.907	3.005 ± 0.900	3.000 ± 0.932	0.971
Smoking, *n* (%)	48 (11.85%)	13 (3.21%)	35 (8.64%)	0.950
Drinking alcohol, *n* (%)	50 (12.35%)	14 (3.46%)	36 (8.89%)	0.921
Diabetes, *n* (%)	47 (11.60%)	8 (1.98%)	39 (9.63%)	0.092
Hypertension, *n* (%)	96 (23.70%)	21 (5.19%)	75 (18.52%)	0.107

*Note:* Bold indicates a statistically significant difference.

Abbreviations: Alb, Albumin; ALT, Alanine Aminotransferase; AST, Aspartate Aminotransferase; BMI, Body Mass Index; GGT, Gamma‐Glutamyl Transferase; Glu, Glucose; HDL‐C, High‐Density Lipoprotein Cholesterol; LDL‐C, Low‐Density Lipoprotein Cholesterol; TC, Total Cholesterol; TG, Triglycerides; UA, Uric Acid; WBC, White Blood Cell Count.

Regarding clinical laboratory examinations, the lymphocyte count in the SIBO− group was 1.94 ± 0.59 × 10^9^/L, whereas the SIBO+ group had a count of 1.80 ± 0.61 × 10^9^/L, with a *p*‐value of 0.035, indicating a significantly lower lymphocyte count in the SIBO+ group compared to the SIBO− group. In addition, creatinine levels were significantly higher in the SIBO+ group than in the SIBO− group (*p* = 0.035). Other blood indicators, such as neutrophils, hemoglobin, platelet count, and monocytes, showed no significant differences between the groups. Liver function, renal function, and metabolic markers (including Alb, UA, ALT, AST, GGT, HDL‐C, TC, TG, and LDL‐C) also did not show significant differences.

### 4.2 Analysis of Correlation Between SIBO and Clinical Symptoms

To explore the potential association between SIBO and digestive symptoms, we administered a questionnaire to patients and compared the differences in digestive symptoms between the SIBO− and SIBO+ groups (Table 2). The results revealed that the incidence of acid reflux and diarrhea was significantly different in the SIBO+ group compared to the SIBO− group, with *p*‐values of 0.011 and 0.009, respectively, indicating statistically significant differences.

**TABLE 2:** Correlation analysis of SIBO and clinical symptoms.

Symptom	Total	SIBO−	SIBO+	*p*
Sleep disorders, *n* (%)	65 (16.05%)	18 (4.44%)	47 (11.60%)	0.516
Bloating, *n* (%)	54 (13.33%)	18 (4.44%)	36 (8.89%)	0.291
Acid reflux, *n* (%)	80 (19.75%)	31 (7.65%)	49 (12.10%)	**0.011**
Abdominal pain, *n* (%)	28 (6.91%)	9 (2.22%)	19 (4.69%)	0.570
Nausea, *n* (%)	29 (7.16%)	10 (2.47%)	19 (4.69%)	0.368
Diarrhea, *n* (%)	46 (11.36%)	20 (4.94%)	26 (6.42%)	**0.009**
Constipation, *n* (%)	61 (15.06%)	14 (3.46%)	47 (11.60%)	0.413
Belching, *n* (%)	48 (11.85%)	13 (3.21%)	35 (8.64%)	0.732
Early satiety, *n* (%)	20 (4.94%)	6 (1.48%)	14 (3.46%)	0.783
Anorexia, *n* (%)	13 (3.21%)	3 (0.74%)	10 (2.47%)	0.738
Exhaust more, *n* (%)	72 (17.78%)	20 (4.94%)	52 (12.84%)	0.929

*Note:* Bold indicates statistical significance.

However, other digestive symptoms such as sleep disorders (*p* = 0.516), abdominal distension (*p* = 0.291), nausea (*p* = 0.368), constipation (*p* = 0.413), hiccups (*p* = 0.732), early satiety (*p* = 0.783), loss of appetite (*p* = 0.738), and fatigue (*p* = 0.929) did not show significant differences between the two groups.

In summary, the findings suggest that SIBO may be associated with the development of acid reflux and diarrhea, while its relationship with other digestive symptoms appears to be weak or non‐significant.

### 4.3 Risk Factors for SIBO

To better understand the risk factors associated with SIBO, we constructed a regression model that included both well‐established risk factors and variables that were found to be significant in univariate logistic regression analyses (Table 3). This methodology aimed to provide a more thorough assessment of potential predictors for SIBO. The results of the regression analysis revealed that the number of lymphocytes was significantly associated with the occurrence of SIBO. Univariate analysis showed that an increase in lymphocyte count was associated with a significantly higher risk of SIBO (OR = 1.519, 95% CI: 1.037–2.226, *p* = 0.032). This association was further confirmed in multivariate analysis (OR = 1.567, 95% CI: 1.036–2.371, *p* = 0.034). In addition, diarrhea was negatively associated with SIBO in univariate analyses (OR = 0.446, 95% CI: 0.239–0.831, *p* = 0.011), and this relationship remained significant in multivariate analyses (OR = 0.465, 95% CI: 0.227–0.954, *p* = 0.037, Table 3).

**TABLE 3:** Univariate and multivariate logistic regression analyses for factors associated with SIBO.

Factor	Univariate analysis				Multivariate analysis			
	OR	95% CI		*p*	OR	95% CI		*p*
Lymphocyte (×10^9^/L)	1.519	1.037	2.226	**0.032**	1.567	1.036	2.371	**0.034**
Creatinine (umol/L)	0.985	0.971	0.999	**0.037**	0.987	0.973	1.001	0.078
Acid regurgitation, *n* (%)	0.523	0.316	0.867	**0.012**	0.598	0.336	1.067	0.082
Diarrhea, *n* (%)	0.446	0.239	0.831	**0.011**	0.465	0.227	0.954	**0.037**

*Note:* Bold indicates statistical significance.

**TABLE 4:** Logistic regression analysis to assess the correlation between SIBO and lymphocytes in different subgroups.

Factor		Univariate analysis				Multivariate analysis			
		OR	95% CI		*p*	OR	95% CI		*p*
Sex, *n* (%)	Male	2.045	1.138	3.673	**0.017**	2.086	1.007	4.324	**0.048**
	Female	1.423	0.775	2.615	0.255	1.606	0.724	3.561	0.244
Age, year	≥ 40	1.998	1.208	3.306	**0.007**	2.215	1.222	4.013	**0.009**
	< 40	1.306	0.593	2.874	0.507	0.593	0.142	2.468	0.472
BMI, kg/m^2^	≤ 23.9	2.298	1.057	4.995	**0.036**	2.744	1.175	6.410	**0.020**
	> 23.9	1.120	0.615	2.038	0.712	1.155	0.612	2.181	0.656

*Note:* Bold indicates statistical significance.

Abbreviation: BMI, Body Mass Index.

However, creatinine and acid reflux showed more complex relationships with SIBO. In particular, acid reflux did not remain significant in multivariate analyses, which suggests that the relationship between acid reflux and SIBO may be influenced by other factors or that the effects of acid reflux on SIBO are more complex.

### 4.4 Logistic Regression Analysis Assessed the Association Between Lymphocytes and SIBO in Different Subgroups

To determine whether lymphocytes have different effects in various populations, we conducted a subgroup analysis, stratifying the population by sex, age, and BMI. Our findings suggest that the association between lymphocyte count and SIBO varies significantly across these subgroups (Table 4).

Specifically, men showed a higher risk of SIBO in univariate analysis (OR = 2.045, 95% CI: 1.138–3.673, *p* = 0.017), and this relationship was further confirmed in multivariate analysis (OR = 2.086, 95% CI: 1.007–4.324, *p* = 0.048). For age, the risk of SIBO in individuals aged ≥ 40 years was significantly higher in univariate analysis (OR = 1.998, 95% CI: 1.208–3.306, *p* = 0.007), and this association remained statistically significant in multivariate analysis (OR = 2.215, 95% CI: 1.222–4.013, *p* = 0.009). Additionally, according to China's obesity standards (Wang et al. 2021), a BMI > 23.9 indicates overweight or obesity, while a BMI ≤ 23.9 represents normal weight or underweight. Patients with a BMI ≤ 23.9 kg/m^2^ exhibited a higher risk of SIBO, as evidenced in both univariate analysis (OR = 2.298, 95% CI: 1.057–4.995, *p* = 0.036) and multivariate analysis (OR = 2.744, 95% CI: 1.175–6.410, *p* = 0.020).

These results suggest that lymphocyte count is a significant predictor of SIBO in middle‐aged and older men with a BMI ≤ 23.9 kg/m^2^.

### 4.5 Diagnostic Value of Lymphocytes in Different Subgroups of SIBO

To evaluate the predictive power of the lymphocyte and SIBO relationship across different subgroups (sex, age, and BMI), ROC curves were plotted for each population group.

**FIGURE 1:**
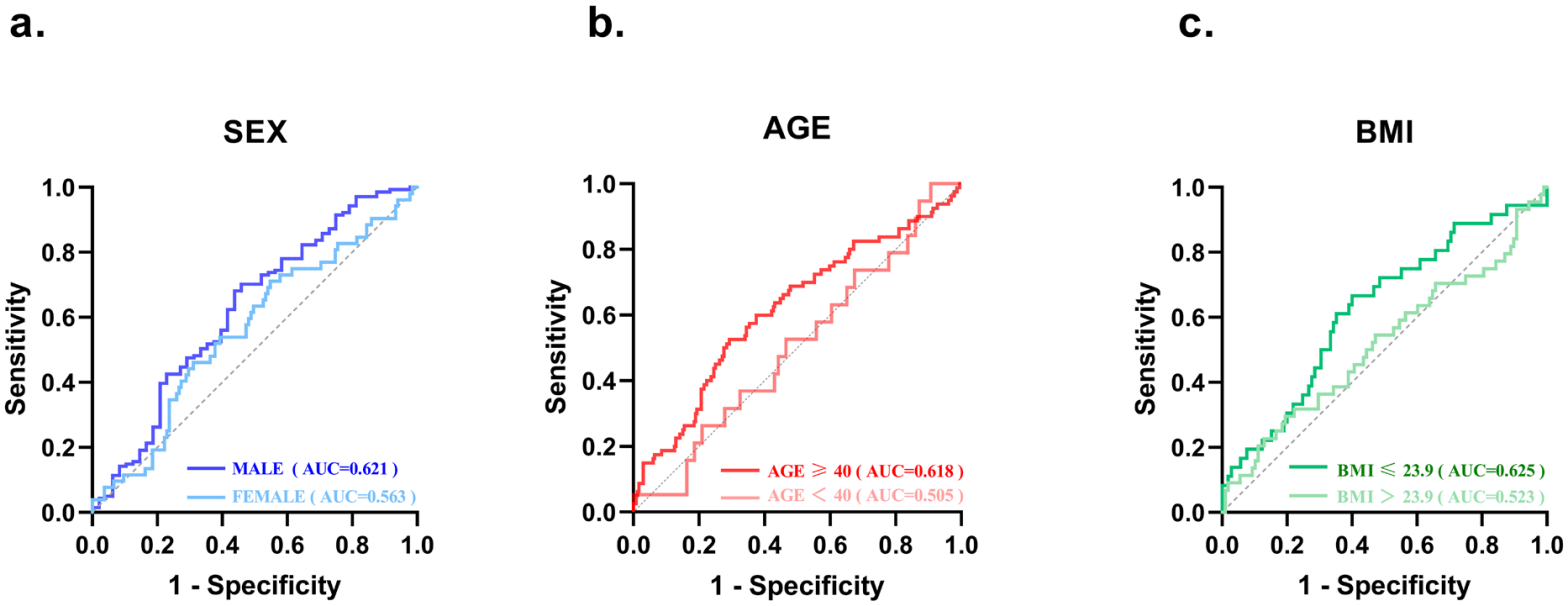
Comparison of receiver operating characteristic (ROC) curves of different populations. These results demonstrate the ability of lymphocytes to predict SIBO. AUC represents the area under the curve.

In the gender subgroup (Figure 1a), the AUC was 0.621 in males, which was significantly higher than the AUC of 0.563 in females. This suggests that lymphocyte count is more predictive of SIBO in males. In the age subgroup (Figure 1b), individuals aged ≥ 40 years had an AUC of 0.618, significantly higher than the AUC of 0.505 in those aged < 40 years. This indicates that lymphocyte count is a stronger predictor of SIBO in the middle‐aged and elderly population. In the BMI subgroup (Figure 1c), the AUC for the BMI ≤ 23.9 kg/m^2^ group was 0.625, which was significantly higher than the AUC of 0.523 in the BMI > 23.9 group. This suggests that lymphocyte count is a more reliable predictor of SIBO in individuals with a lower BMI.

Taken together, these results highlight significant differences in the predictive value of lymphocytes for SIBO risk across different populations, with stronger predictive ability observed in middle‐aged and older adults, males, and patients with low BMI.

## 5 Composition of Gut Microbiota

### 5.1 Basic Features of Gut Microbiota in SIBO

30 patients were selected using propensity score matching with a one to two ratio, including 10 SIBO− and 20 SIBO+ patients. Due to quality control issues, one stool sample was excluded, leaving a final analysis of 29 patients, consisting of 10 SIBO− and 19 SIBO+ patients.

**FIGURE 2:**
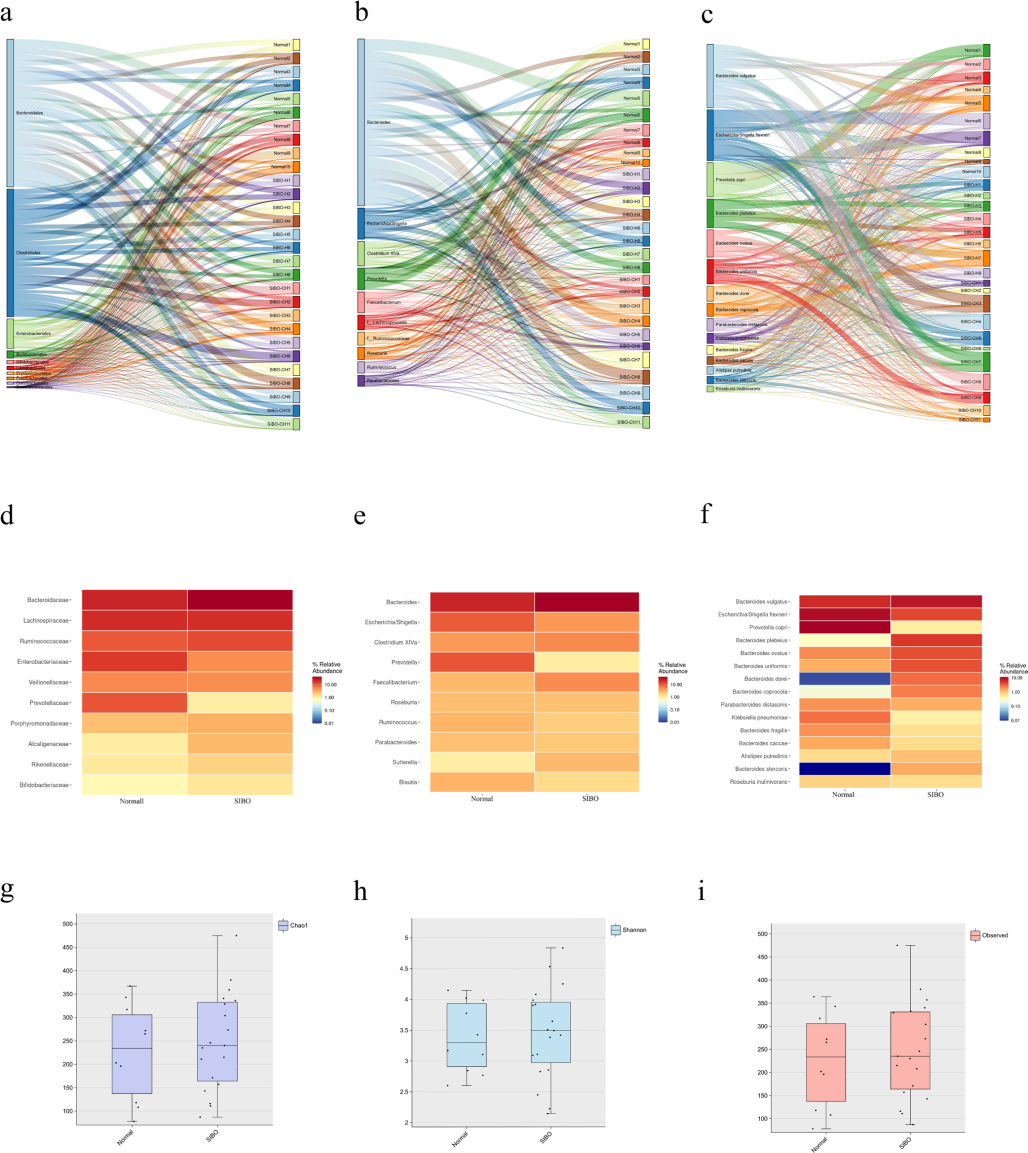
Distribution of intestinal microbiome in a single sample at the phylum level (a), top 10 genera by abundance (b), and top 15 species by abundance (c). (d) Distribution of intestinal microbiome at the phylum level for each of the three groups, (e) top 10 genera by abundance, and (f) top 15 species by abundance. (g) Alpha diversity based on the Chao1 index between groups; (h) alpha diversity based on the Shannon index between groups; (i) alpha diversity based on the Observed index between groups.

We summarized the microbial distribution characteristics of the different samples. At the phylum level (Figure 2a,d), Bacteroidales were more prevalent in the SIBO‐negative group, while Enterobacteriales were significantly increased in the SIBO‐positive group, suggesting a shift in microbial composition in SIBO patients. At the genus level (Figure 2b,e), Bacteroides and Faecalibacterium showed a significant decrease in the SIBO group, while the abundance of potential pathogenic bacteria, such as *Escherichia/Shigella*, was significantly higher in the SIBO‐positive patients. At the species level (Figure 2c,f), *Bacteroides dorei* increased significantly in the SIBO group, while *Prevotella copri* and *Klebsiella pneumoniae* were notably reduced, further highlighting the microbial shifts that accompany SIBO.

These findings suggest that SIBO is associated with significant alterations in gut microbiota composition, including both the increase in potentially pathogenic bacteria and a decrease in beneficial species.

### 5.2 Intra‐Group Diversity Analysis

We analyzed the diversity of gut microbiota in SIBO‐negative and SIBO‐positive patients. Our results indicated no significant difference in the α‐diversity between the two groups, as measured by the Chao1 index and Shannon diversity index (Figure 2g,h,i). The findings suggest that SIBO does not significantly alter the intra‐group microbial diversity.

**FIGURE 3:**
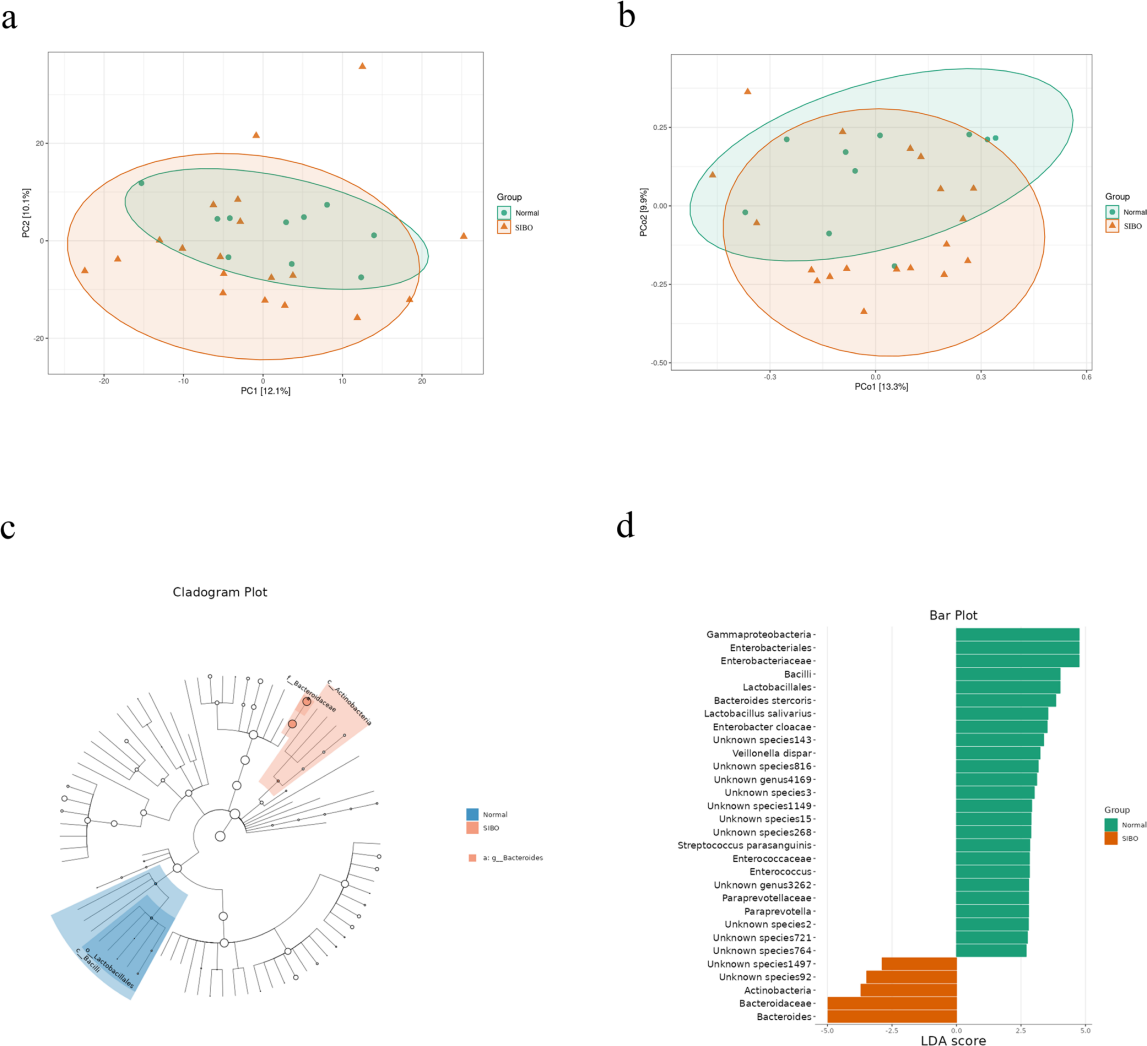
(a) Bray‐Curtis‐based β‐diversity analysis; (b) Bray‐Curtis‐based PCoA (principal coordinates analysis); (c, d) LEfSe analysis showing phylogenetic differences between groups. Only results with LDA > 2 are shown.

To further assess the complexity of species distribution between the SIBO− and SIBO+ groups, we conducted beta‐diversity analysis. Dimensionality reduction through PCA and PCoA highlighted significant differences in the fecal microbiota composition between the two groups. Then, the PCA (*p* = 0.040) and PCoA (*p* = 0.047) analyses indicated that SIBO significantly influenced the overall distribution of the gut microbiota (Figure 3a,b).

The results suggest that while SIBO may not affect microbial diversity within each group, it does significantly influence the overall composition and distribution of gut microbiota.

### 5.3 LefSe Analysis of Microbial Biomarkers

To further explore the differential microbiota associated with SIBO, we performed LEfSe analysis of the gut microbiota in SIBO− and SIBO+ patients. The results revealed several key microbial taxa that were significantly enriched in the SIBO‐positive group. These taxa primarily included Bacteroides‐related bacteria, such as Bacteroidaceae, Bacteroides, and Actinobacteria (Figure 3c,d). The increased abundance of these bacteria in the SIBO‐positive group indicates their potential involvement in the development of small intestinal bacterial overgrowth, possibly through mechanisms like microbial imbalance or dysbiosis in the gut.

### 5.4 Fundamental Features of Gut Microbiota Metabolites in SIBO

**FIGURE 4:**
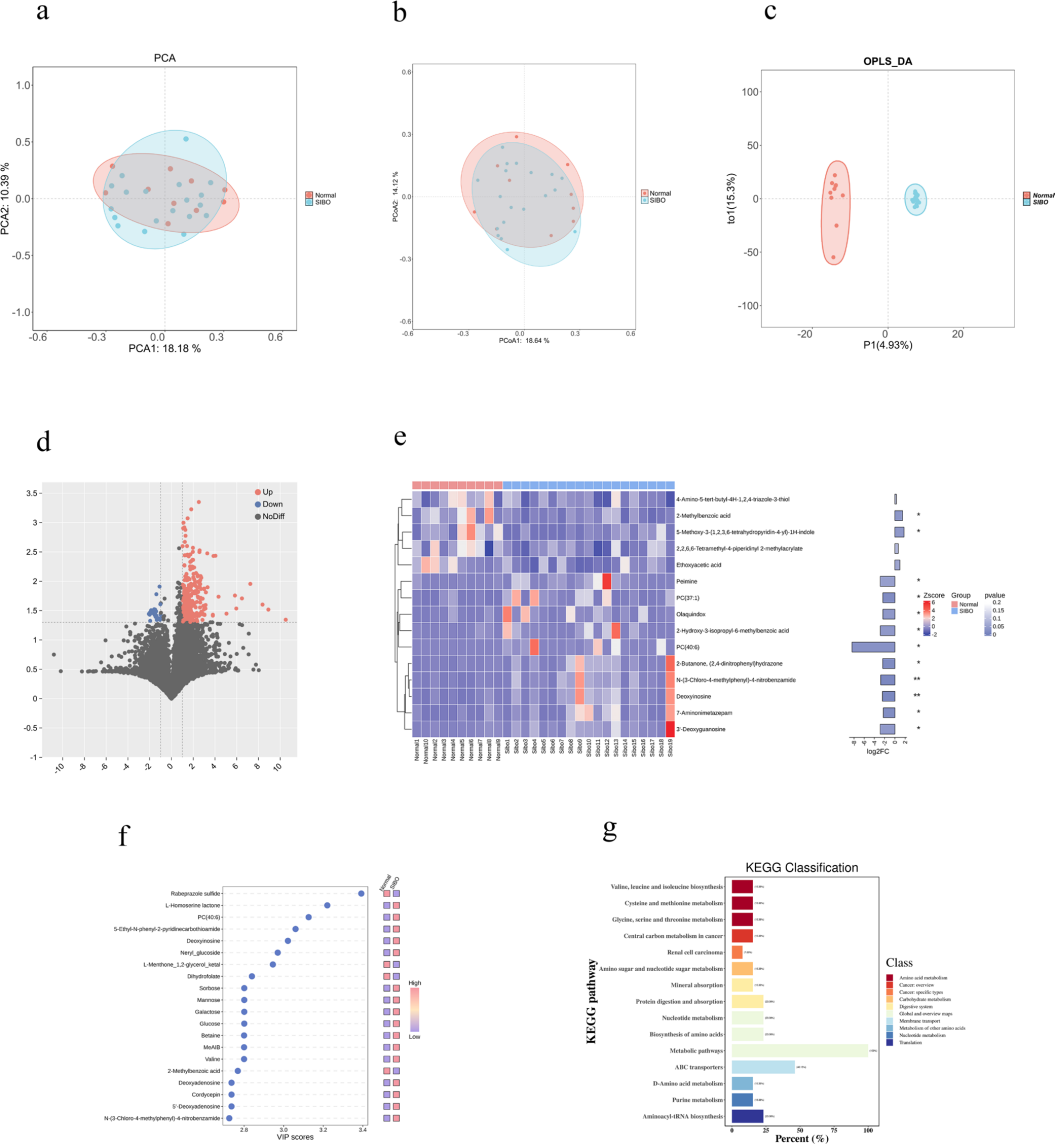
Metabolomic analysis of gut microbiota metabolites in each group. (a) PCA based on intestinal microbiome metabolites; (b) PCoA plot based on intestinal microbiome metabolites; (c) OPLS‐DA analysis based on intestinal microbiome metabolites. Volcano plots of differential metabolites among the three groups: (d) Healthy control group vs. SIBO+; (e) Heatmap of differential metabolites. (f) VIP scores of differential metabolites and corresponding heatmap. (g) Annotated map of KEGG pathways for differentially abundant metabolites. *p < 0.05; **p < 0.01.

To further investigate the differences in gut microbiota‐derived metabolites among the SIBO population, we conducted PCA (Figure 4a) and PCoA (Figure 4b) analyses. These methods revealed some variations in gut microbial metabolites between SIBO+ patients and healthy controls. However, no significant differences were found between the two groups. Notably, OPLS‐DA analysis (Figure 4c) demonstrated distinct metabolic profiles of the gut microbiota between SIBO+ patients and healthy controls. These findings highlight significant alterations in the gut microbiota metabolic profile in SIBO‐positive patients.

To further explore the differential metabolites, we conducted volcano map analysis to compare the normal population and SIBO+ patients (Figure 4d). The results revealed 329 different metabolites between the normal and SIBO+ groups, of which 301 were upregulated and 28 downregulated. This indicates that SIBO is associated with significant metabolic alterations in the gut. Additionally, we visualized the metabolites with the most significant differences using heat maps and VIP value maps (Figure 4e,f). Among the metabolites with the greatest differences, PC (40:6) showed the most significant change, while Rabeprazole sulfide was the most unusual metabolite identified in this analysis.

To explore the broader implications of these differential metabolites, we annotated the KEGG pathways (Figure 4g). Among the top three enriched pathways were metabolic pathways, ATP‐Binding Cassette (ABC transporters), and biosynthesis of amino acids. These altered pathways suggest that metabolic disruptions in SIBO may affect key biological functions, including nutrient transport and amino acid synthesis, which could provide new insights into the pathophysiological mechanisms of SIBO.

### 5.5 Analysis of the Correlation Between Gut Microbiota and Metabolite Profiles

**FIGURE 5:**
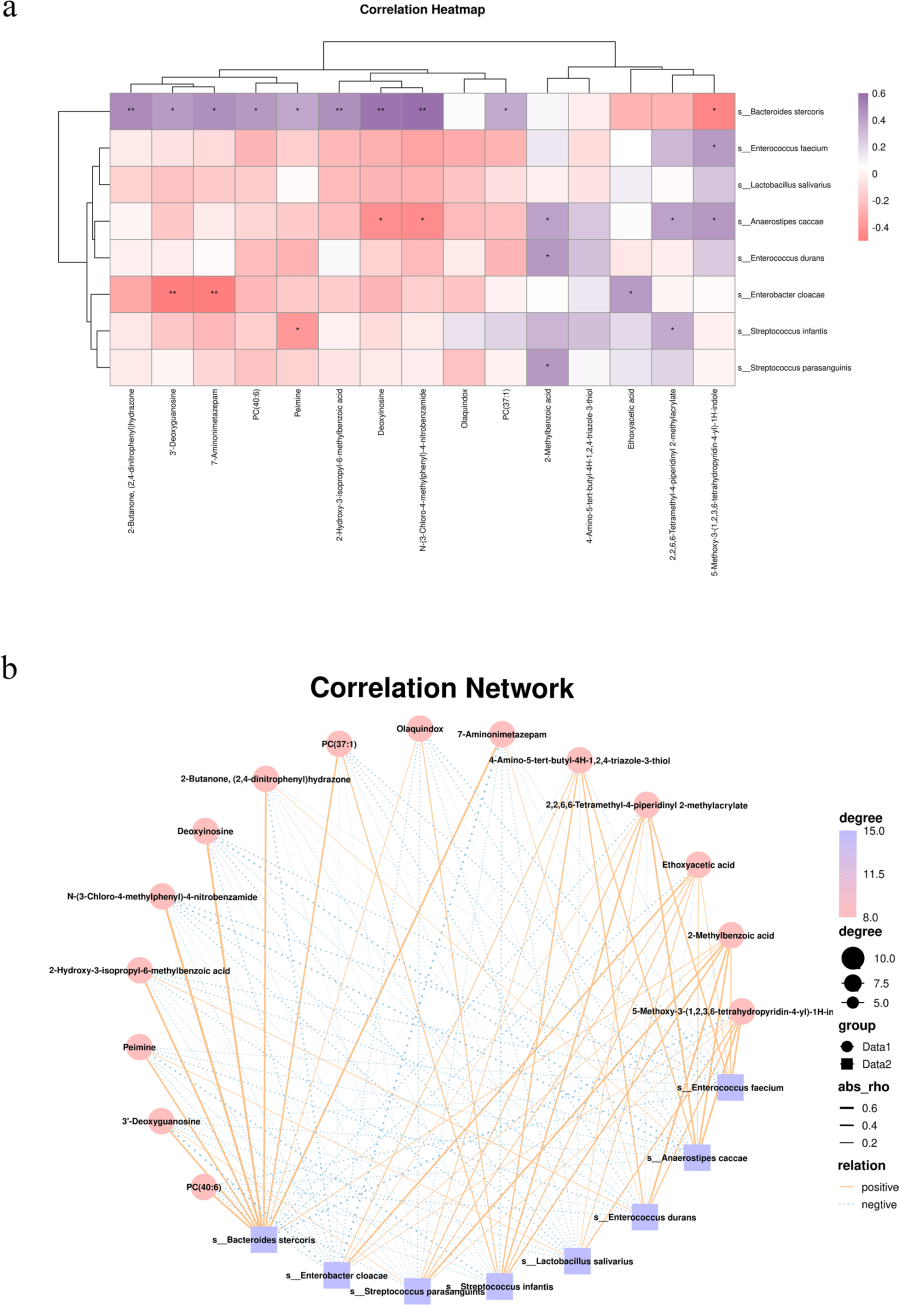
(a) Heatmap of fecal microorganisms associated with metabolites. The rows represent differentially abundant microbes, and the correlation coefficients are shown in the legend on the right. Purple indicates a positive correlation, and pink indicates a negative correlation. (b) Network map of fecal microbiota associated with metabolites. The rows represent different microbial species, and the legend on the right displays the correlation coefficients: Solid orange lines indicate positive correlations, while dashed blue lines represent negative correlations. *p < 0.05, **p < 0.01.

By using Spearman's correlation analysis, we explored the relationship between differential gut microbiota and metabolites in SIBO patients (Figure 5a,b). Then, the results showed significant correlations between metabolic changes and the abundance of several bacterial taxa. *Bacteroides stercoris* exhibited the most significant correlation with several metabolites, including: 2‐Butanone, (2,4‐dinitrophenyl)hydrazone, 2‐hydroxy‐3‐isopropyl‐6‐methylbenzoic acid, deoxyinosine, and methylbenzoic acid N‐(3‐Chloro‐4‐methylphenyl)‐4‐nitrobenzamide. These metabolites showed strong associations with *Bacteroides stercoris* , suggesting that this bacterium may play a pivotal role in modulating metabolic processes in the small intestine during SIBO. In addition, *Anaerostipes caccae* was found to be closely related to two of these metabolites, further highlighting the complex interactions between gut microbes and their metabolic byproducts in the context of SIBO.

### 5.6 Correlation Analysis of Intestinal Flora, Metabolism and Serological Indexes

**FIGURE 6:**
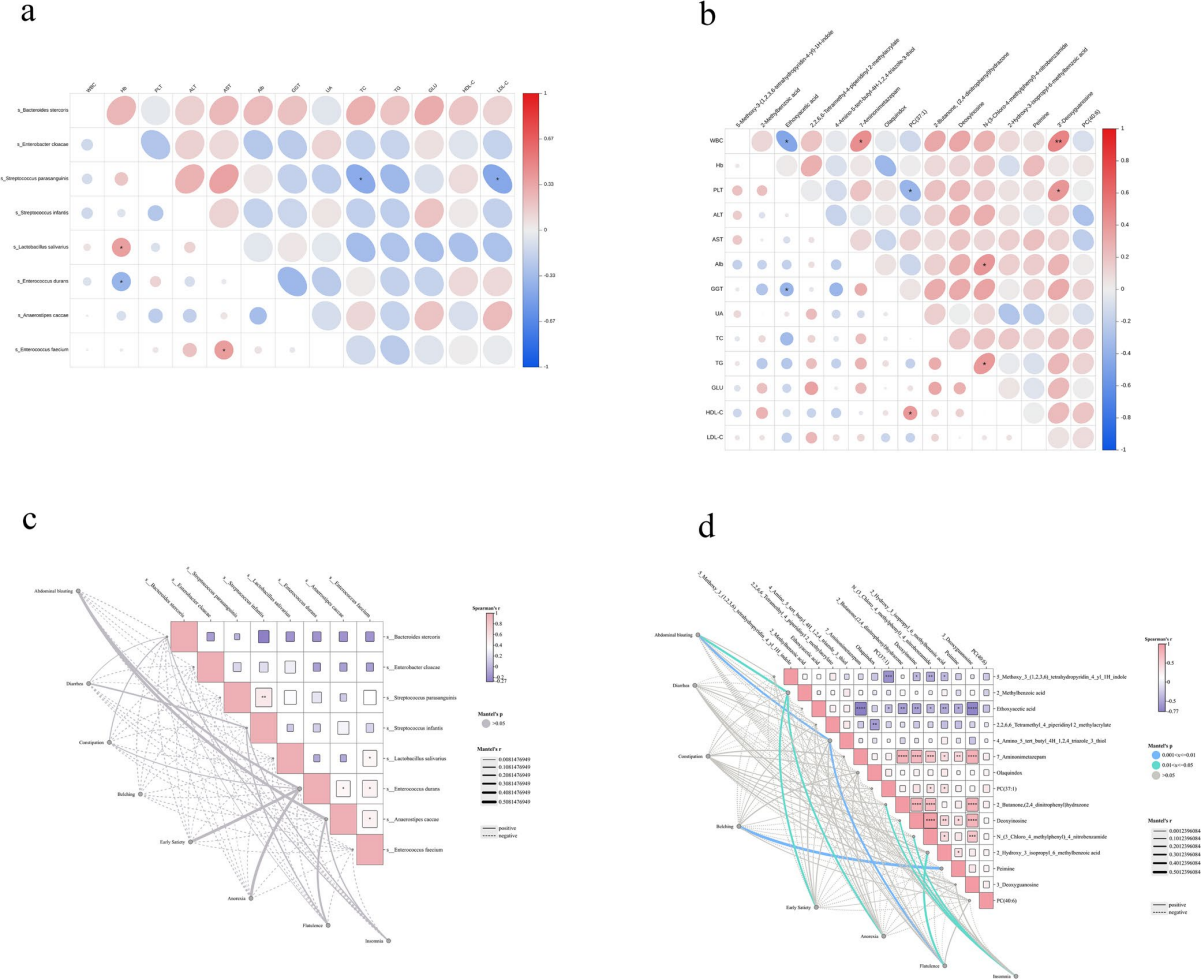
Serological markers and intestinal differential flora: (a) Heatmap of the correlation between serological markers and intestinal differential flora, where blue indicates a negative correlation and red indicates a positive correlation. (b) Heatmap of the metabolic profile of the intestinal differential flora, with blue representing a negative correlation and red representing a positive correlation. Clinical symptoms and intestinal differential flora: (c) Correlation between clinical symptoms and intestinal differential flora. (d) Heatmap of clinical symptoms and intestinal differential flora, with a solid line indicating a positive correlation and a dashed line indicating a negative correlation. *p < 0.05, **p < 0.01, ***p < 0.001, ****p < 0.0001.

The relationship between gut microbiota and serological metabolism was explored through a correlation analysis between microbial characteristics and various serological markers (Figure 6a,b).

Microbiota and Metabolic Markers: *Lactobacillus salivarius* showed a significant positive correlation with hemoglobin concentration. This suggests that *L. salivarius* may play a role in iron metabolism or erythropoiesis, potentially influencing red blood cell production or iron homeostasis. *Enterococcus durans* exhibited a significant negative correlation with γ‐glutamyltransferase, an enzyme linked to liver function. This implies a possible protective role of *E. durans* against liver damage or dysfunction. *Anaerostipes caccae* was positively correlated with HDL‐C, suggesting that this bacterium may contribute to improved lipid metabolism or cardiovascular health by influencing lipid profiles. *Enterococcus faecium* showed a significant positive correlation with LDL‐C, indicating a potential involvement in lipid regulation, particularly in relation to cholesterol metabolism.

Bacterial Metabolites and Serological Markers: Further investigation into the correlation between bacterial metabolites and serological indicators revealed several notable associations: 4‐Amino‐5‐tert‐butyl‐4H‐1,2,4‐triazole‐3‐thiol exhibited a significant positive correlation with platelet count. This may suggest a potential link between this metabolite and blood clotting or inflammatory processes. N‐(3‐Chloro‐4‐methylphenyl)‐4‐nitrobenzamide showed a significant positive correlation with hemoglobin concentration, indicating a possible involvement in red blood cell production or oxygen transport. 2‐Hydroxy‐3‐isopropyl‐6‐methylbenzoic acid was positively correlated with HDL cholesterol, reinforcing the hypothesis that microbial metabolites influence lipid metabolism, particularly the regulation of healthy cholesterol levels. 2‐Methylbenzoic acid was negatively correlated with γ‐glutamyltransferase, suggesting a potential protective or regulatory role in liver function, possibly indicating a role in mitigating liver stress or damage.

### 5.7 Correlation Analysis of Intestinal Flora and Metabolism With Clinical Symptoms

Spearman correlation and meta‐analysis were used to identify associations between intestinal microbiota and metabolites with clinical symptoms (Figure 6c,d). The analysis revealed correlations between certain microbiota (e.g., *Bacteroides stercoris* , *Enterococcus faecium* , *Lactobacillus salivarius* ) and gastrointestinal symptoms (e.g., bloating, diarrhea, constipation, belching, and early satiety). However, these associations were not statistically significant, suggesting that while changes in gut microbiota may be involved in the pathogenesis of these symptoms, they do not exhibit specific or consistent associations at a statistically significant level.

In terms of microbiota metabolites, several metabolites were found to be significantly associated with clinical symptoms. For bloating, metabolites such as 4‐Amino‐5‐tert‐butyl‐4H‐1,2,4‐triazole‐3‐thiol and 2‐methylbenzoic acid showed significant correlations. For insomnia, metabolites including N‐(3‐Chloro‐4‐methylphenyl)‐4‐nitrobenzamide, 2‐Hydroxy‐3‐isopropyl‐6‐methylbenzoic acid, and Deoxyinosine were significantly associated. Network analysis further illustrated the complex and interconnected relationships between the microbiome and gastrointestinal symptoms, reinforcing the idea that the gut microbiota may play an important role in gastrointestinal health. However, the observed correlations highlight the complexity and need for further research to determine the precise mechanisms and specificity of these associations.

## 6 Discussion

The balance of gut microbiota is crucial for maintaining normal digestion, immune function, and overall health. Under normal conditions, the microbial community in the gut interacts synergistically with the host's immune, digestive, and nervous systems through complex mechanisms. However, when the gut microbiota becomes imbalanced, certain bacterial populations may proliferate excessively. In particular, bacteria that typically reside in the large intestine may translocate to the small intestine, leading to SIBO (Maeda and Murakami 2023). SIBO itself can further exacerbate gut microbiota imbalances, creating a vicious cycle. The overgrowth of bacteria results in the fermentation of incompletely digested food, producing gas and increasing the gas burden within the gut. This excess gas may have detrimental effects on the intestinal barrier, potentially increasing intestinal permeability and heightening the risk of pathogenic microorganism invasion (Vaga et al. 2020). Thus, the correlation between SIBO and gut microbiota is bidirectional, with an imbalance in the gut microbiota contributing to the development of SIBO, while SIBO itself worsens microbiota imbalances, leading to a self‐perpetuating cycle. Our study delves into this intricate relationship, aiming to reveal the profound interactions between SIBO and gut microbiota.

To identify clinical hematobiochemical markers associated with Small Intestinal Bacterial Overgrowth (SIBO), we conducted a retrospective study focusing on SIBO‐positive populations. The results revealed significant differences in lymphocyte counts between these individuals and controls. This association highlights the regulatory role of the immune system in maintaining gut microbiome balance. Lymphocytes, as a critical component of the immune system, play an essential role in intestinal immune surveillance and in maintaining the balance of gut microbiota (Mowat and Agace 2014). The mechanisms underlying this relationship can be described as follows: First, lymphocytes, especially T cells and B cells, are the primary mediators of the intestinal immune response. Gut‐associated lymphoid tissues, such as Peyer's patches, are rich in immune cells responsible for recognizing and eliminating pathogenic microorganisms from the gut (Hooper et al. 2012). When immune system function is impaired, it can lead to a breakdown of the intestinal barrier, which may provide an opportunity for the development of SIBO. Second, in cases of immune dysfunction, the ability of lymphocytes to recognize and clear overgrown bacteria from the small intestine is compromised. This failure allows excessive bacterial proliferation, triggering the onset of SIBO (Kayama et al. 2020). Additionally, bacterial overgrowth leads to the production of gases and metabolites that stimulate inflammation in the gut. In a chronic inflammatory state, an overactive immune response can further disrupt the gut microbiota, contributing to the persistence of SIBO (Allaire et al. 2018). Finally, immune tolerance is crucial for maintaining a balanced intestinal microecological environment. The immune tolerance mechanisms in the gut help prevent the immune system from overreacting to normal flora. Disruption of these mechanisms or dysfunction in immune cells can lead to gut dysbiosis, fostering the development of SIBO (Graham and Xavier 2023). To conclude, our findings indicate a strong association between SIBO and alterations in lymphocyte numbers, which may reflect changes in immune system function, emphasizing the importance of immune regulation in preventing SIBO.

In addition, we investigated the potential of lymphocytes as a predictive marker for SIBO in specific populations, and our results demonstrated that lymphocytes have strong predictive value in non‐obese middle‐aged and elderly men. The possible mechanisms behind this association are as follows: First, the immune system undergoes gradual decline with age, a phenomenon known as immunosenescence. This age‐related decline results in a reduction in both the number and function of lymphocytes, particularly the immune response capabilities of T cells and B cells (Saldana‐Morales et al. 2021). Lymphocytes play a crucial role in intestinal immune surveillance by maintaining the integrity of the intestinal barrier and regulating the balance of gut microbiota. When lymphocyte function is weakened, intestinal immune surveillance becomes impaired, leading to bacterial imbalance and an increased risk of SIBO (Recaldin et al. 2024). Second, altered immune tolerance in middle‐aged and elderly individuals may also contribute to SIBO. Immune tolerance refers to the ability of the immune system to recognize and coexist with the normal gut flora. When immune tolerance mechanisms fail, the immune system may not effectively differentiate between beneficial and harmful bacteria, resulting in dysbiosis and a higher risk of SIBO (Shirafkan et al. 2024). Additionally, gender differences in immune response may influence the susceptibility to SIBO. Men are more affected by chronic low‐grade inflammation than women. Chronic inflammation weakens lymphocyte function, disrupts immune responses, and compromises the intestinal barrier, thus promoting the growth of pathogenic bacteria. As a result, men's immune systems may be more vulnerable to dysfunction caused by chronic inflammation, increasing their likelihood of developing SIBO (Zimmermann and Curtis 2019). Moreover, studies suggest that there are distinct differences in the gut microbiota between men and women, with men's microbiota potentially being more sensitive to changes in immune system function. Immune decline in middle‐aged and elderly men may make their gut microbiota more vulnerable to reduced lymphocyte function, thereby promoting the development of SIBO (Thapar et al. 2020). In summary, the relationship between lymphocyte levels and SIBO in non‐obese middle‐aged and elderly men involves a combination of factors such as immune system decline, altered immune tolerance, compromised intestinal barrier function, and chronic low‐grade inflammation. Immune aging, sex differences, and immune dysfunction appear to be key contributors to this relationship. Therefore, immune function, lymphocyte levels, and immune system decline in non‐obese middle‐aged and elderly men together enhance the predictive value of lymphocytes for identifying individuals at high risk for SIBO. This provides an important clinical biomarker for identifying and monitoring individuals at increased risk for SIBO.

Moreover, the clinical symptoms of SIBO remain inconclusive, and detailed studies have been conducted to identify which specific digestive symptoms are significantly associated with the condition. Our results demonstrate a significant correlation between acid reflux, diarrhea, and SIBO. The underlying mechanisms may include the following: First, SIBO leads to dysregulation of the small intestine microbiota, disrupting the balance of normal gut flora. Under normal conditions, small intestinal peristalsis helps push food and bacterial residues toward the large intestine. However, when bacterial overgrowth occurs, intestinal motility is impaired, causing food and gas to remain in the small intestine for extended periods (Delbaere et al. 2023). This dysmotility not only exacerbates bacterial proliferation but may also further impair intestinal function, worsening SIBO. As a result, gas accumulation in the small intestine may force some of it to flow back into the stomach. This gas buildup can lead to abnormal gastric acid secretion and delay gastric emptying, increasing stomach pressure and causing gastric contents to regurgitate into the esophagus, thereby resulting in acid reflux symptoms (Efremova et al. 2024). Moreover, abnormal gastric acid secretion not only disrupts the digestion and absorption of nutrients but may also alter the microbial community in the stomach, exacerbating gastrointestinal discomfort. Acid reflux may also occur due to interactions between stomach acid and small intestinal bacteria, which affect the normal migration of stomach contents, creating a vicious cycle that worsens SIBO and gastroesophageal reflux disease (Mishima and Ishihara 2020). Additionally, bacterial imbalances caused by SIBO can deplete nutrients from food and interfere with the gut's normal absorption processes. Typically, the gut microbiota supports nutrient digestion and absorption, but when bacteria overgrow, they may compete with intestinal cells for nutrients, impairing the absorption of water, salts, and essential nutrients, ultimately contributing to diarrhea (Yang et al. 2020). Lastly, SIBO may compromise the intestinal barrier, increasing contact between intestinal epithelial cells, bacteria, and toxins. This triggers local inflammatory responses, which can lead to increased intestinal permeability, commonly referred to as “leaky gut.” This allows partially digested food, toxins, and bacteria to enter the bloodstream, further activating the immune response and stimulating intestinal water secretion, ultimately resulting in diarrhea (Zhang et al. 2021). In summary, the significant association between acid reflux, diarrhea, and SIBO can be explained by mechanisms such as intestinal flora imbalance, disrupted motility, gas accumulation, and delayed gastric emptying. These factors collectively contribute to the development and exacerbation of SIBO, offering valuable insights into the understanding and management of SIBO‐related symptoms in clinical practice.

The colon microbiota is the most diverse and complex part of the intestinal microbiome, playing crucial roles in metabolism and immune modulation. The primary groups of colonic bacteria include *Firmicutes*, *Actinobacteria*, *Pseudomonas*, *Clostridium*, and *Verrucomicrobia*, with *Firmicutes* and *Bacteroides* dominating, accounting for about 90% of the human gut microbiota (Rinninella et al. 2019). In the jejunum, bacterial populations are lower, around 10^5^ CFU/mL, primarily composed of *Bacteroides*, *Lactobacillus*, and *Streptococcus*. In the ileum, bacterial concentrations increase to 10^8^ CFU/mL, with *Bacteroides*, *Clostridium*, *Enterococcus*, *Lactobacillus*, *Veillonella*, and *Enterobacteriaceae* being predominant (Gałecka et al. 2018). These bacteria play a vital role in breaking down dietary fiber and complex carbohydrates, producing short‐chain fatty acids (SCFAs). These SCFAs provide energy for intestinal epithelial cells, maintaining intestinal barrier integrity and regulating immune function. Research has shown that patients with SIBO exhibit distinctive microbiota characteristics in the small intestine. In individuals with bacterial counts ≥ 10^3^ to < 10^5^ CFU/mL and ≥ 10^5^ CFU/5 mL, there is a noticeable decrease in the α‐diversity of duodenal microbiota, with a significant increase in the relative abundance of *Escherichia* and *Klebsiella* (*p* < 0.0001 and *p* = 0.0018, respectively). This shift leads to reduced microbial network connectivity. Additionally, metabolic pathways such as carbohydrate fermentation, hydrogen production, and hydrogen sulfide generation are enhanced in subjects with ≥ 10^3^ CFU/mL, and these metabolic alterations are closely associated with clinical symptoms (Leite et al. 2024). Our study aligns with these findings, revealing that SIBO patients show an increased abundance of *Escherichia* and *Klebsiella* in the gut. Interestingly, we also found that in SIBO‐positive patients, the colon microbiota is characterized by a significant abundance of *Bacteroidetes* and *Actinobacteria*. *Bacteroides* plays a critical role in the breakdown of dietary fiber and complex carbohydrates, leading to the production of SCFAs such as acetic acid, propionic acid, and butyric acid. These metabolites are essential for maintaining intestinal health, reinforcing the intestinal barrier, and modulating the immune system (Pan et al. 2023). Furthermore, the development of intestinal intraepithelial lymphocytes (IELs) has been shown to depend on *Bacteroidetes*, with the enzyme β‐hexosaminase, conserved across *Bacteroidetes*, promoting the differentiation of IELs. Specific T cells can recognize multiple symbiotic bacteria and trigger regulatory immune responses within the gut (Bousbaine et al. 2022). *Actinobacteria* is a Gram‐positive, non‐motile bacterial phylum, typically forming branched structures, and is widely distributed in soil, decaying organic matter, and the human intestine and skin. *Actinomyces* species contribute to the global carbon cycle by decomposing plant biomass (Lewin et al. 2016; Dinesh et al. 2017). Moreover, *Actinobacteria* are associated with various diseases and may influence the development of neurological conditions, such as Parkinson's disease, through the brain‐gut axis (Li et al. 2021).

The metabolite enrichment of intestinal flora in SIBO is primarily involved in carbohydrate fermentation, hydrogen and hydrogen sulfide production, and organic acid metabolism. The overgrowth of bacteria in the small intestine leads to the fermentation of undigested carbohydrates, such as dietary fiber and sugars, resulting in the production of SCFAs like acetic acid, butyric acid, and propionic acid. These SCFAs contribute to the energy supply of intestinal cells and help maintain the intestinal barrier function. However, excessive fermentation products can lead to gas accumulation, particularly hydrogen and hydrogen sulfide. This buildup not only contributes to symptoms such as bloating and abdominal pain but also interferes with normal intestinal motility, which exacerbates the pathological condition of SIBO (Guo et al. 2024). Additionally, SIBO triggers the overproduction of organic acids, such as lactic acid and acetic acid, which reduce the intestinal pH, altering the intestinal environment. This change can affect the activity of digestive enzymes and inhibit the growth of certain beneficial bacteria, thereby exacerbating intestinal dysfunction and malabsorption. Hydrogen sulfide, a toxic gas produced by sulfur‐reducing bacteria during fermentation, can damage intestinal epithelial cells. It has strong toxicity and can induce local inflammatory responses, further compromising the intestinal barrier function and increasing the risk of intestinal permeability (Vaga et al. 2020). These metabolites not only disrupt the gut's microbiological balance but are also closely associated with the hallmark symptoms of SIBO, such as bloating, abdominal pain, and diarrhea. Understanding how these metabolic pathways interact with symptom‐generating mechanisms offers new clinical insights and identifies potential therapeutic targets. Intervening in these metabolic pathways—such as reducing the production of harmful gases, regulating acidic metabolites, or improving the balance of intestinal flora—could effectively alleviate the clinical symptoms of SIBO and provide novel strategies for treatment.

Furthermore, we conducted an in‐depth analysis to explore the correlation between serological metabolism, intestinal flora, and microbial characteristics. Our findings revealed significant associations between specific bacteria and various metabolic indicators. *Lactobacillus salivarius* showed a strong positive correlation with hemoglobin concentration, suggesting that this bacterium may promote hemoglobin synthesis by modulating the gut environment or enhancing iron absorption and utilization. *Enterococcus durans* was found to be negatively correlated with gamma‐glutamyltransferase, potentially reflecting its protective role in regulating liver metabolism and oxidative stress. Furthermore, *Anaerostipes caccae* exhibited a significant positive correlation with HDL‐C, indicating that it may regulate lipid metabolism via short‐chain fatty acid production. In contrast, *Enterococcus faecium* was positively correlated with LDL‐C, which could be linked to its influence on cholesterol absorption or metabolism. We also investigated the correlation between bacterial metabolites and serological indicators. Our analysis revealed that metabolites play a significant role in regulating the host's metabolic state. Specifically, N‐(3‐Chloro‐4‐methylphenyl)‐4‐nitrobenzamide was positively correlated with hemoglobin, further supporting the idea that metabolites may influence erythropoiesis. On the other hand, 2‐Hydroxy‐3‐isopropyl‐6‐methylbenzoic acid showed a strong positive correlation with HDL‐C, likely reflecting its role in lipid metabolism pathways. Additionally, 2‐Methylbenzoic acid was negatively correlated with GGT, suggesting that it may act as a protective agent by mitigating oxidative stress or liver metabolic disturbances. These results highlight the complex and intertwined relationship between gut microbiota and host metabolism.

In addition, we conducted an in‐depth exploration of the clinical symptoms, intestinal flora, and metabolites of the patients. However, we were unable to identify specific flora or metabolites associated with acid reflux and diarrhea. There are several possible explanations for this finding. First, the significant individual variability among the study samples, such as differences in diet, lifestyle, and other personal factors, may have masked the potential associations between specific microbiota or metabolites and these symptoms. Second, the occurrence of acid reflux and diarrhea is likely influenced by the combined action of multiple microorganisms and metabolites, rather than being attributable to a single bacterial group or metabolite. The complex interactions within the gut microbiome may not be easily captured by univariate analysis, thus making it challenging to identify significant correlations.

The study has several limitations. First, the diagnosis of SIBO was based on breath tests, which are less sensitive and specific compared to other diagnostic methods. While small intestine sampling could provide more accurate results, it is too invasive for routine use. Additionally, traditional bacterial culture techniques are limited in detecting anaerobic bacteria, which may lead to an underestimation of bacterial diversity in the gut. Second, the small sample size and lack of population diversity further limit the statistical power of the study. Finally, the relationship between the gut microbiota and SIBO is complex, and while correlation studies can identify associations, they cannot establish causality. Furthermore, research into the underlying mechanisms remains insufficient. These limitations highlight the need for improved diagnostic tools, more robust analytical methods, and larger, more diverse sample sizes in future studies to enhance the quality and reliability of research in this area.

## 7 Conclusion

Changes in lymphocyte count demonstrate strong predictive power for the screening and diagnosis of SIBO, particularly in middle‐aged and elderly non‐obese men, highlighting its potential clinical utility. The primary clinical symptoms of SIBO include diarrhea and acid reflux, which may be linked to an imbalance in the intestinal microbiota, intestinal dysfunction, and gastrointestinal motility disorders. Our study identified Bacteroides as the most characteristic intestinal microbiota in SIBO patients, suggesting that this microbiota plays an important role in SIBO, particularly in the regulation of metabolism and the maintenance of intestinal barrier integrity. Furthermore, the metabolic changes associated with SIBO involve several key metabolic pathways. In future studies, targeting these biomarkers and metabolic pathways may offer novel therapeutic strategies for the personalized treatment of SIBO, providing more accurate and effective treatment options for patients.

## Author Contributions

All authors made substantial contributions to the reported work, including but not limited to the conception and design of the study, data acquisition, analysis and interpretation of results, and drafting or critically revising the manuscript. Each author gave final approval of the manuscript for publication, agreed upon the journal to which the article was submitted, and is accountable for all aspects of the work.

## Ethics Statement

This study was conducted in accordance with the principles outlined in the Declaration of Helsinki and received approval from the Ethics Committee of Beijing Shijitan Hospital, affiliated with Capital Medical University (approval number: sjtky11‐1x‐2022 [63]). Informed consent was obtained from all participants prior to their inclusion in the study.

## Consent

We obtained informed consent from patients or their immediate family members after informing them of the purpose and significance of the study.

## Conflicts of Interest

The authors declare no conflicts of interest.

## Data Availability

All clinical data from this study are retained by the authors to ensure patient privacy. If raw data is required, it can be obtained by emailing the corresponding author with a valid request. The gut microbiome datasets analyzed in this study have been submitted to NCBI, with the dataset identifier [PRJNA1269711].
